# Supplementary material for: Intrathecal activation of CD8+ memory T cells in IgG4‐related disease of the brain parenchyma
Source: EMBO Mol Med. 2021 Jul 13;13(8):e13953. doi: 10.15252/emmm.202113953 (PMC8350898; doi:10.15252/emmm.202113953)
Supplement: Supplementary file 1 — Appendix [file EMMM-13-e13953-s002.zip › emmm202113953-sup-0005-FigS5.pdf]

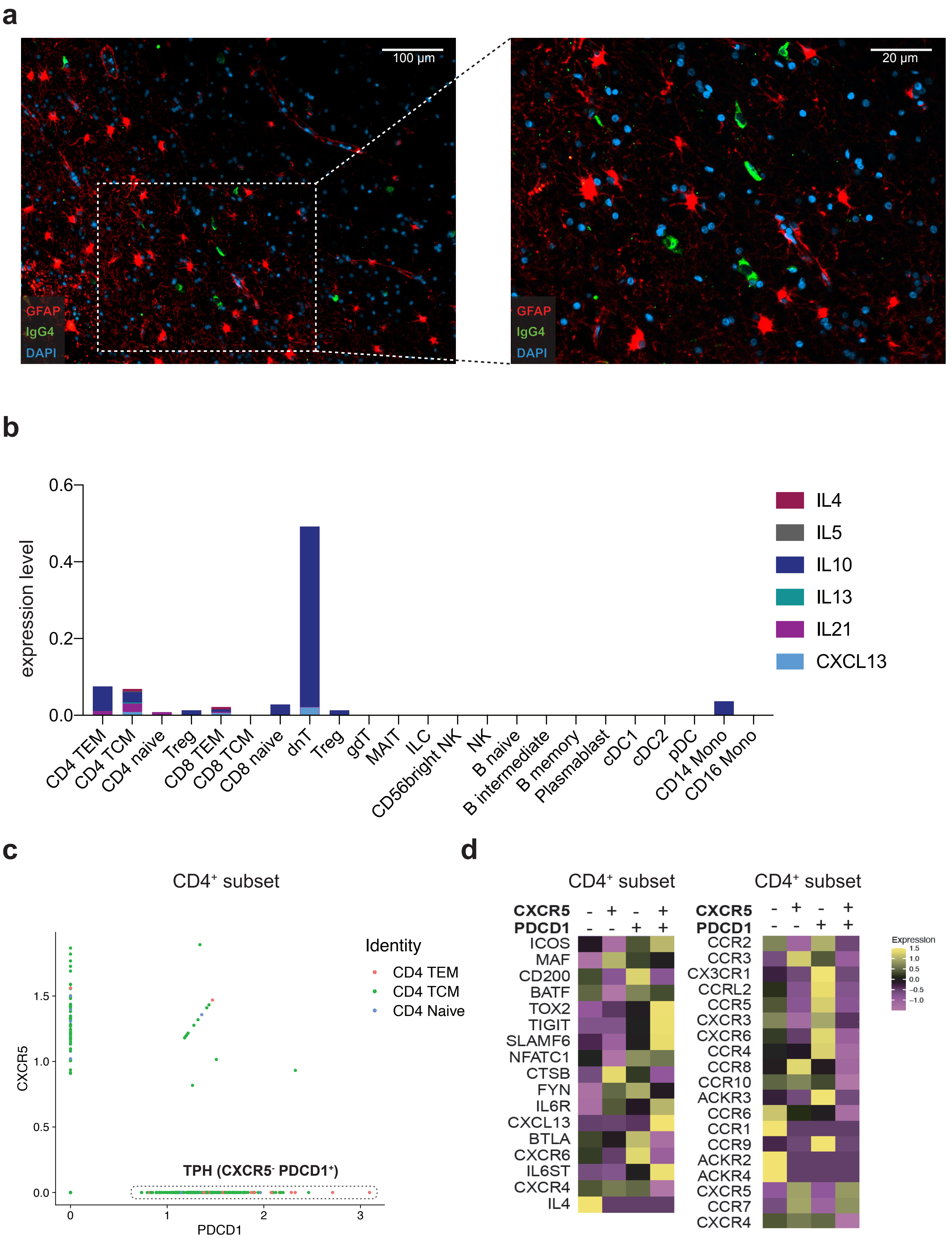
a
100 µm
20 µm
GFAP
IgG4
DAPI
b
expression level
0.6
0.4
0.2
0.0
IL4
IL5
IL10
IL13
IL21
CXCL13
CD4 TEM
CD4 TCM
CD4 naive
Treg
CD8 TEM
CD8 TCM
CD8 naive
dnT
Treg
gdT
MAIT
ILC
CD56bright NK
NK
B naive
B intermediate
B memory
Plasmablast
cDC1
cDC2
pDC
CD14 Mono
CD16 Mono
c
CD4⁺ subset
Identity
CD4 TEM
CD4 TCM
CD4 Naive
CXCR5
PDCD1
TPH (CXCR5⁻ PDCD1⁺)
d
CD4⁺ subset
CXCR5 - + - +
PDCD1 - - + +
ICOS
MAF
CD200
BATF
TOX2
TIGIT
SLAMF6
NFATC1
CTSB
FYN
IL6R
CXCL13
BTLA
CXCR6
IL6ST
CXCR4
IL4
CD4⁺ subset
CXCR5 - + - +
PDCD1 - - + +
CCR2
CCR3
CX3CR1
CCRL2
CCR5
CXCR3
CXCR6
CCR4
CCR8
CCR10
ACKR3
CCR6
CCR1
CCR9
ACKR2
ACKR4
CXCR5
CCR7
CXCR4
Expression
1.5
1.0
0.5
0.0
−0.5
−1.0
