# Supplementary figures and images for: Intrathecal activation of CD8+ memory T cells in IgG4‐related disease of the brain parenchyma
Source: EMBO Mol Med. 2021 Jul 13;13(8):e13953. doi: 10.15252/emmm.202113953 (PMC8350898; doi:10.15252/emmm.202113953)

**a**

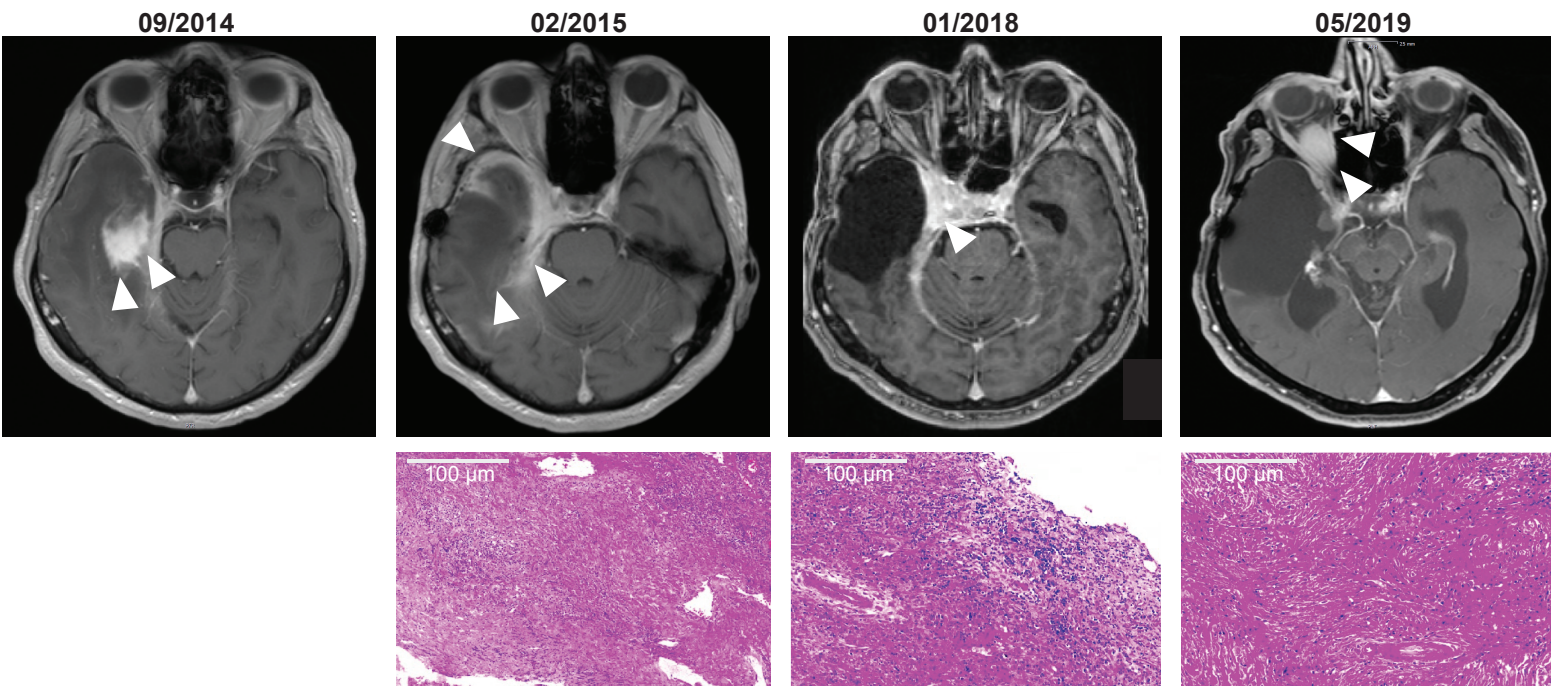

**b**

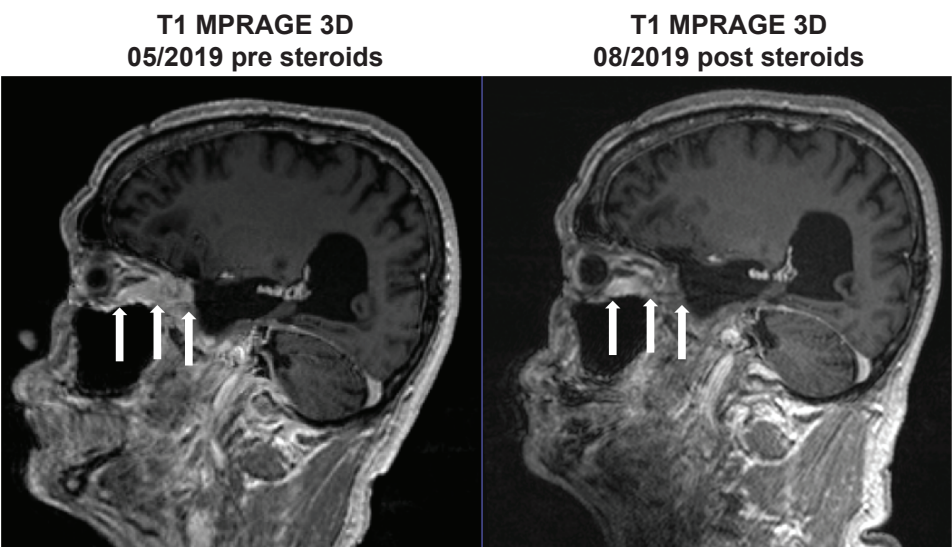

**c**

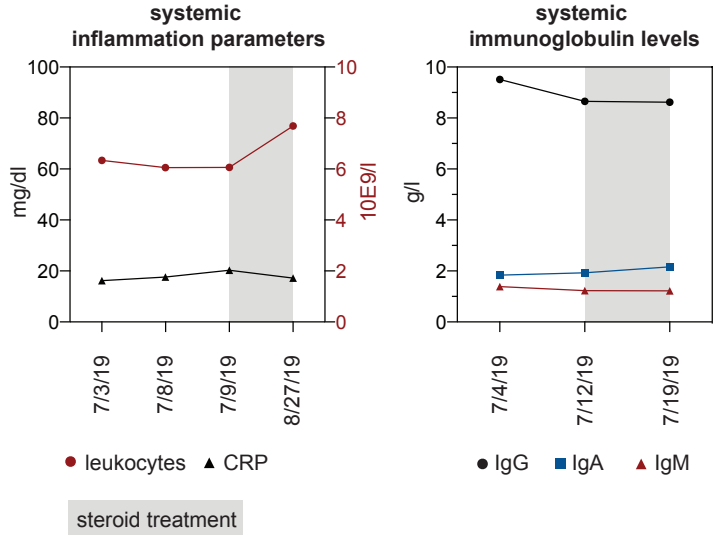

**d**

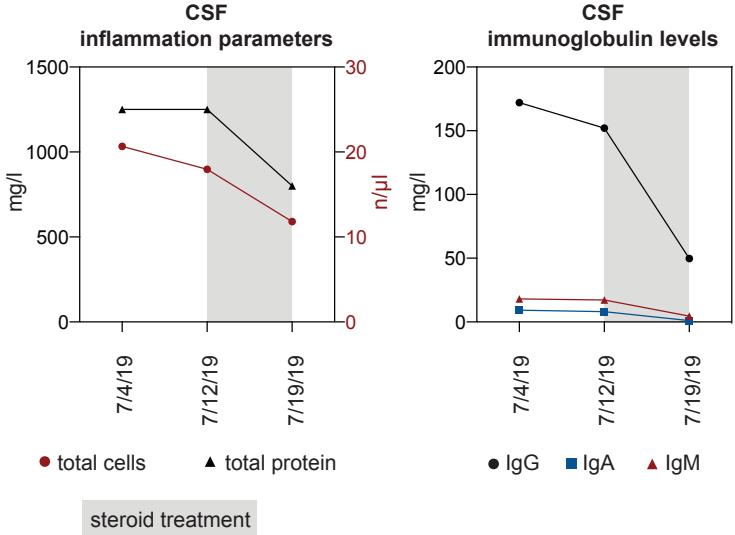

Supplement: Supplementary file 1 — Appendix [file EMMM-13-e13953-s002.zip › emmm202113953-sup-0001-FigS1.pdf]

**a****H&E**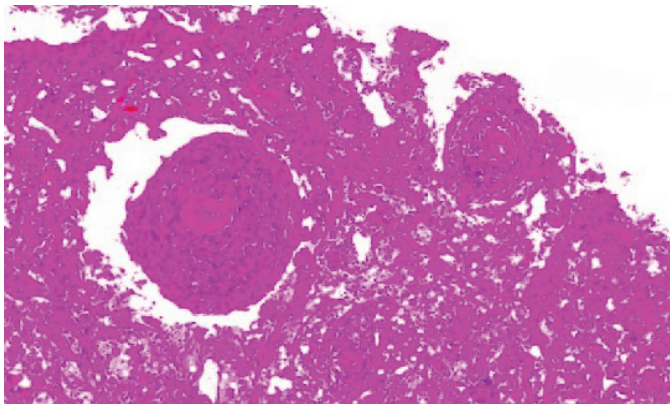25μm**b****IgG4**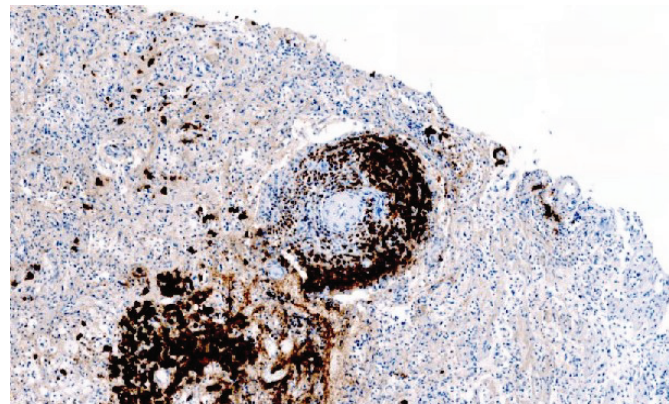25μm**c****H&E**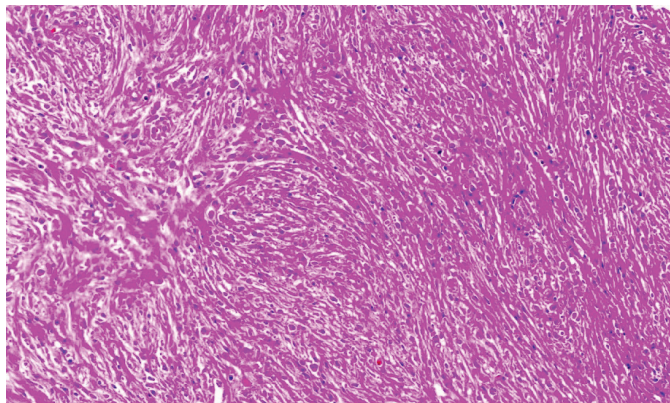100μm**d****EvG**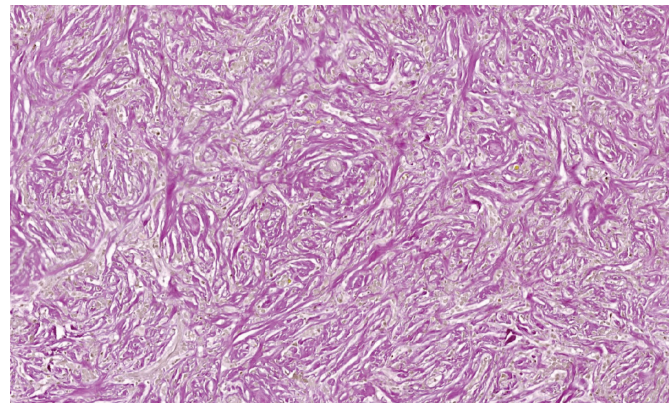100μm

Supplement: Supplementary file 1 — Appendix [file EMMM-13-e13953-s002.zip › emmm202113953-sup-0002-FigS2.pdf]

**a**

02/2015

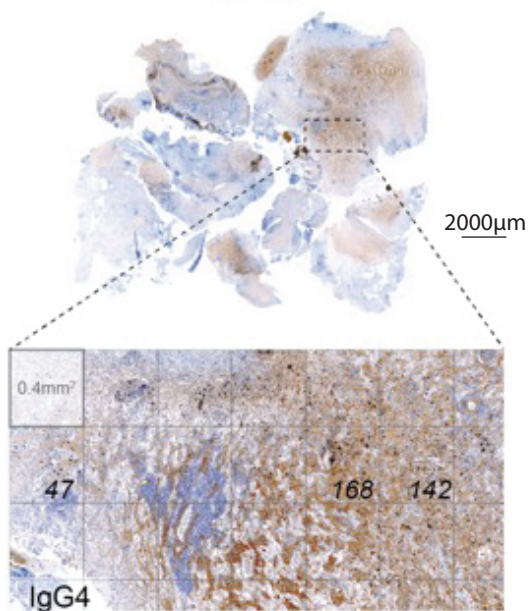

01/2018

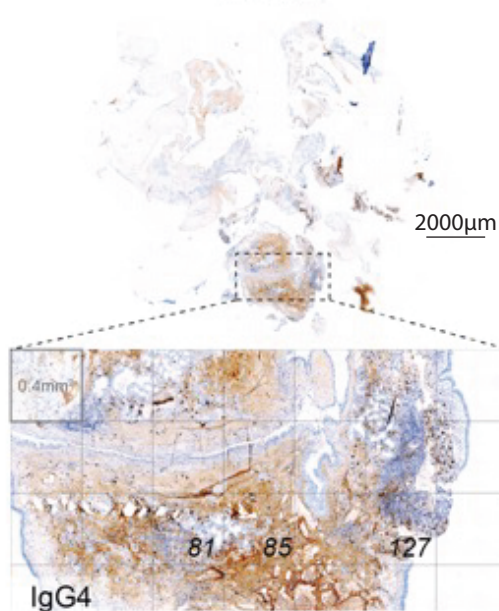

05/2019

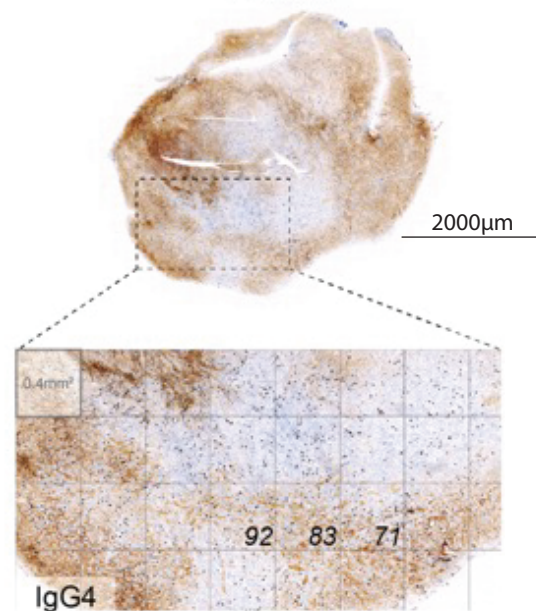**b**

02/2015

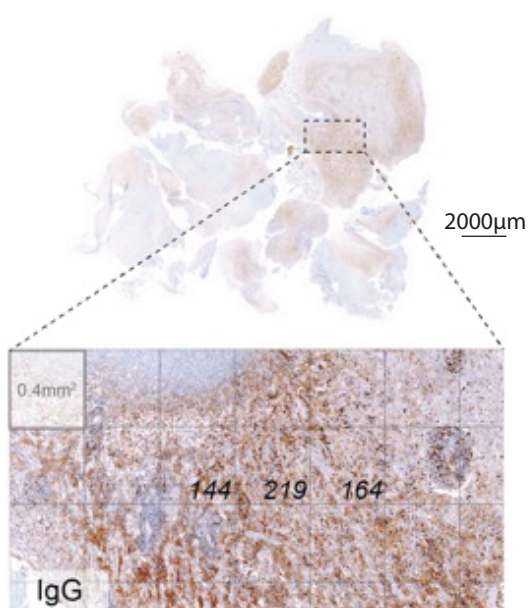

01/2018

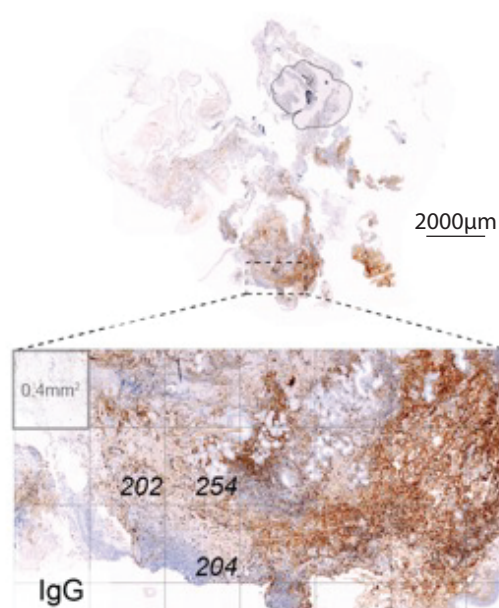

05/2019

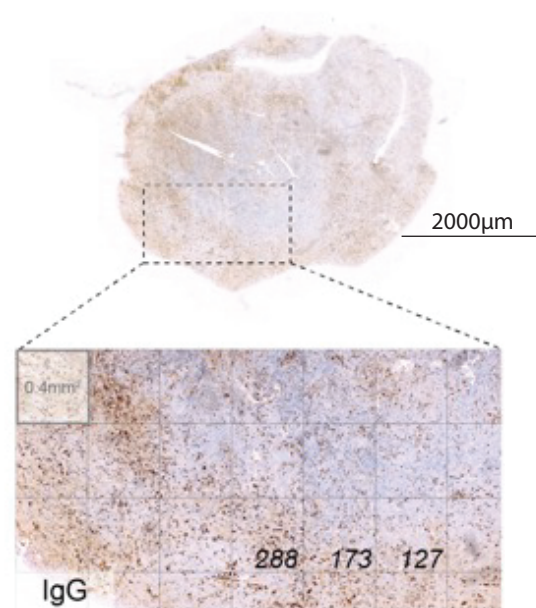**c**

02/2015

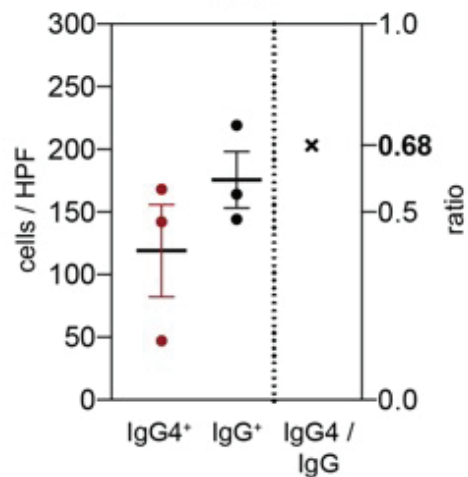

01/2018

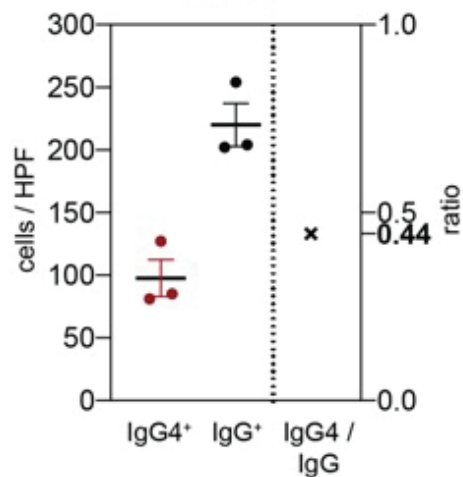

05/2019

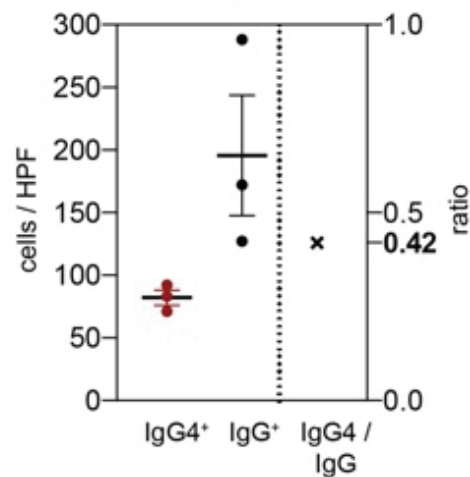

Supplement: Supplementary file 1 — Appendix [file EMMM-13-e13953-s002.zip › emmm202113953-sup-0003-FigS3.pdf]
