# Supplementary material for: Intrathecal activation of CD8+ memory T cells in IgG4‐related disease of the brain parenchyma
Source: EMBO Mol Med. 2021 Jul 13;13(8):e13953. doi: 10.15252/emmm.202113953 (PMC8350898; doi:10.15252/emmm.202113953)
Supplement: Supplementary file 2 — Expanded View Figures PDF [file EMMM-13-e13953-s003.pdf]

Not for Delivery
