# Supplementary material for: Intrathecal activation of CD8+ memory T cells in IgG4‐related disease of the brain parenchyma
Source: EMBO Mol Med. 2021 Jul 13;13(8):e13953. doi: 10.15252/emmm.202113953 (PMC8350898; doi:10.15252/emmm.202113953)
Supplement: Supplementary file 1 — Appendix [file emmm202113953-sup-0004-FigS4.pdf › emmm202113953-sup-0004-FigS4.pdf]

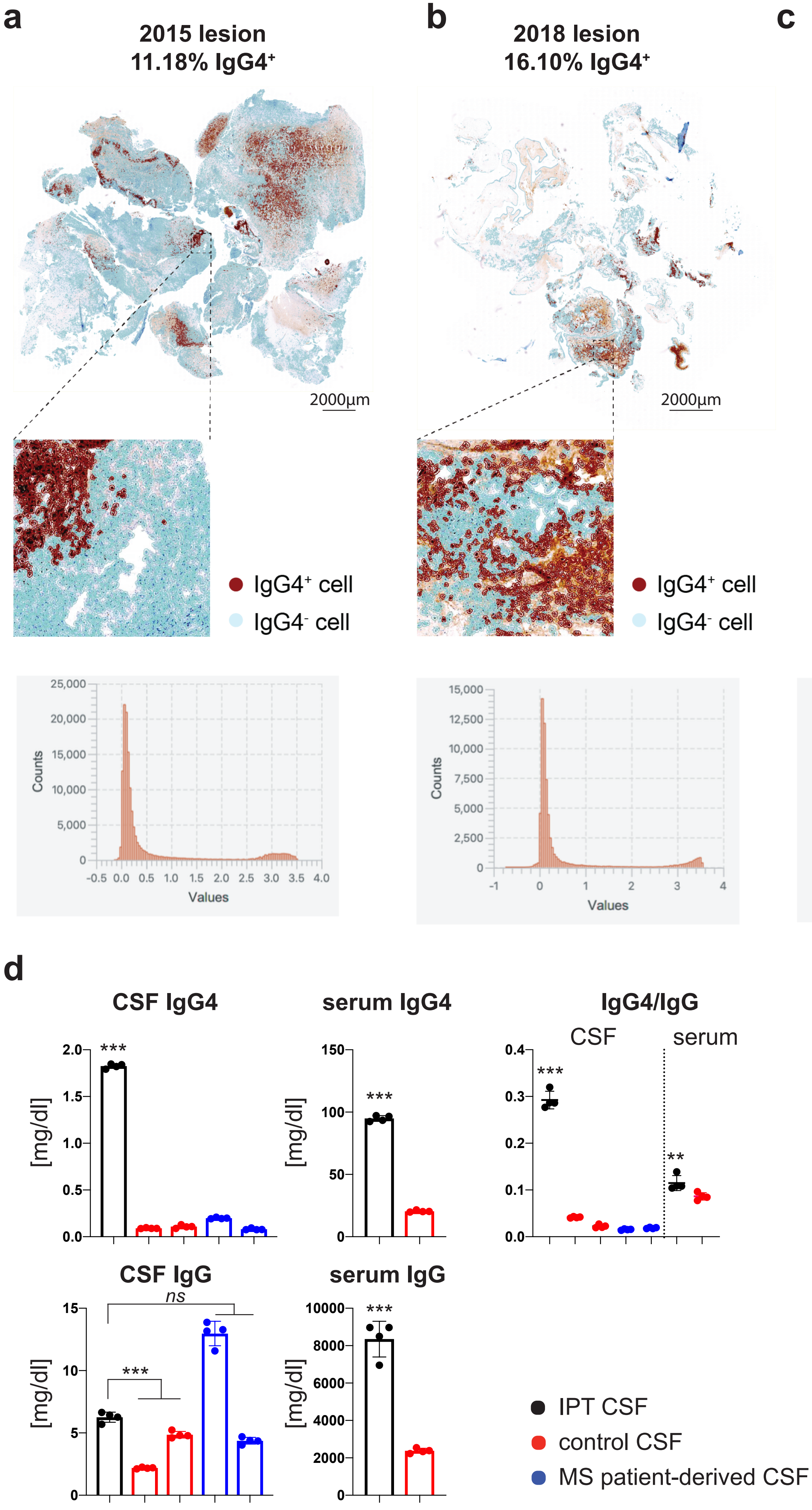

a
2015 lesion
11.18% IgG4+
2000µm
IgG4+ cell
IgG4- cell
Counts
Values
b
2018 lesion
16.10% IgG4+
2000µm
IgG4+ cell
IgG4- cell
Counts
Values
c
2019 lesion
28.07% IgG4+
2000µm
IgG4+ cell
IgG4- cell
Counts
Values
d
CSF IgG4
[mg/dl]
***
serum IgG4
[mg/dl]
***
IgG4/IgG
CSF
serum
***
**
CSF IgG
ns
***
[mg/dl]
serum IgG
***
[mg/dl]
IPT CSF
control CSF
MS patient-derived CSF
